# ChatGPT-Based Chatbot for Help Quitting Smoking via Text Messaging: An Interventional Study

**DOI:** 10.2196/79402

**Published:** 2025-10-10

**Authors:** Lorien C Abroms, Christina N Wysota, Artin Yousefi, Tien-Chin Wu, David A Broniatowski

**Affiliations:** 1 Milken Institute School of Public Health George Washington University Washington, DC United States; 2 Grossman School of Medicine New York University New York, NY United States; 3 School of Engineering and Applied Science George Washington University Washington, DC United States

**Keywords:** ChatGPT, large language model, chatbots, tobacco, smoking cessation, cigarettes, SMS text messaging, artificial intelligence

## Abstract

**Background:**

Large language model chatbots such as ChatGPT may be able to provide support to people who smoke cigarettes and are trying to quit.

**Objective:**

This pilot study examined the feasibility and acceptability of integrating a specialized ChatGPT-based chatbot, BeFreeBot, into a smoking cessation text messaging intervention, BeFree. Chatbot fidelity was also examined.

**Methods:**

Participants who smoked cigarettes in the previous 7 days were recruited from Amazon Mechanical Turk (N=23), enrolled in BeFree, and provided access to BeFreeBot. Surveys were administered at baseline and 4 weeks after enrollment to assess perceptions of BeFreeBot. Computer records of interactions between BeFreeBot and participants were also analyzed to assess participant engagement and adherence of BeFreeBot to its instructions. For the adherence analysis, transcripts were dual coded, and discrepancies were resolved by a third coder.

**Results:**

Most participants (16/23, 70%) texted BeFreeBot with questions or concerns at least once. Participants sent 14.5 (SD 23.6) texts to BeFreeBot on average. Most participants were highly satisfied with BeFreeBot (13/18, 72%) and agreed that it was helpful for quitting (11/19, 58%). They also reported that the BeFreeBot responses were clear and easy to understand (16/17, 94%) and that they trusted responses from BeFreeBot (12/17, 71%). Most participants (17/19, 90%) reported trying to quit smoking for 1 day or longer, and 30% (7/23) self-reported no smoking in the previous 7 days. An analysis of transcripts of BeFreeBot responses (n=328) revealed that BeFreeBot functioned as instructed on most measures, with clear language (328/328, 100%), follow-up questions asked of participants (13/16, 81%), and recommendations to seek out professional counseling (13/16, 81%) or consider the use of Food and Drug Administration–approved medications (eg, nicotine replacement therapy; 14/16, 88%). Responses stayed on the topic of smoking cessation counseling (324/328, 98.8%) and did not include information that contradicted the US Preventive Services Task Force guidelines (328/328, 100%).

**Conclusions:**

A specialized large language model chatbot integrated into an SMS text messaging program and accessed through SMS text message was found to be feasible and acceptable to smokers.

## Introduction

### Background

Tobacco use is the leading preventable cause of death, disability, and disease burden in the United States and worldwide [1]. Approximately 11.6% of adults in the United States are current smokers, and an estimated 70% of smokers want to quit [2]. The earlier in the life course an individual stops smoking, the greater the health benefits are for both the individual and society as a whole [1].

The US Preventive Services Task Force (USPSTF) states that effective smoking cessation treatments should offer counseling with social support and pharmacotherapy except where it is contraindicated (eg, pregnancy) [3]. There is a strong dose-response relationship between the intensity of smoking cessation counseling and its effectiveness, with counseling involving more sessions leading to higher quit rates [3].

Automated smoking cessation programs on mobile phones that use SMS text messaging have been found to be effective for smoking cessation and other health behaviors [4-7]. A recent meta-analysis of SMS text messaging programs for smoking cessation concluded that such programs double abstinence rates [5]. SMS text messaging programs for smoking cessation have been used by millions of smokers trying to quit [4].

Chatbots or computerized conversational agents have the potential to extend the capabilities of SMS text messaging programs and other digital interventions by providing interactive and humanlike personalized counseling on quitting [8-13]. Historically, chatbot conversations have been predetermined based on prewritten statements and if-then rules to create simulated conversations with users. One meta-analysis of chatbots for smoking cessation identified 6 trials of such chatbots and found that chatbot use by smokers was associated with a 66% increase in the odds of quitting [10]. The largest and best designed of these was a study by Perski et al [13] that examined the addition of a chatbot to a smoking cessation app and found that offering the chatbot+app increased engagement and improved smoking cessation–related outcomes over offering the app alone. However, most studies to date are limited by having small sample sizes and are of mixed quality. In addition, the chatbots studied predate the introduction of generative artificial intelligence (AI) chatbots [10-13].

With rapid and ongoing developments in generative AI, including with large language models (LLMs), the natural language capabilities of chatbots have significantly improved. LLM chatbots such as OpenAI’s ChatGPT, Google’s Gemini, and Meta’s Llama allow for open-text queries and provide dynamic, natural language responses that are sensitive to context, nuance, and history and resemble a human conversation [14-16]. In 2025, a total of 34% of American adults and 58% of adults under 30 years of age in the United States, reported that they had used ChatGPT, with use rising from the previous year [17]. On April 1, 2024, the World Health Organization released a web-based chatbot based on OpenAI’s ChatGPT, SARAH (Smart AI Resource Assistant for Health), which aims to assist in smoking cessation and modifying other health behaviors. SARAH, which has been evaluated for the content of its smoking cessation information [18], is available in 8 languages and has been used by over 40,000 people [19].

While these chatbots appear promising, challenges to their effective use may include chatbots providing information that is false or invented (ie, “hallucinated”). To date, a handful of studies have investigated LLM chatbots for smoking cessation [11,18,20,21]. One study used experts to rate motivational messages to quit smoking and found most LLM chatbot messages to be highly rated [21]. Another pilot study predating ChatGPT examined whether an app with an LLM chatbot feature could help in quitting smoking and showed promising results [20]. Finally, a study by our research team found that ChatGPT-based chatbots could provide reliable responses to queries on how to quit smoking [18]. Most responses to queries on quitting smoking had clear language (97.3%) and included a recommendation to seek out professional counseling (80.3%). Less common was the recommendation to consider using nicotine replacement therapy (NRT; 52.7%), provision of information on how to deal with cravings when quitting smoking (44.4%), or the recommendation to consider the use of non-NRT prescription drugs (14.1%). Specific queries that were most challenging for the chatbots included queries on how to quit smoking cold turkey, using gummies, or using hypnosis [18].

As LLM chatbots are increasingly used by the public, it is important to evaluate them as tools for smoking cessation and other types of health behavior change. Studies are needed to evaluate whether the instructions and materials provided to LLM chatbots serve as effective guardrails (eg, can chatbots stay on topic, maintain their roles as counselors, and adhere to prespecified guidelines regardless of the query). In addition, studies are needed that can explore how chatbots can be integrated into existing methods that smokers use for quitting and whether people are willing to engage with them. Ultimately, studies are needed to determine whether LLM chatbots can provide benefits to people trying to quit smoking.

### Objectives

This study aimed to assess the feasibility and acceptability of offering a ChatGPT-based chatbot, BeFreeBot, to smokers in the context of an existing SMS text messaging smoking cessation program, BeFree. Key questions of interest for the study included whether people trying to quit smoking using an SMS text messaging program would engage with a chatbot (feasibility), how they would view the helpfulness of the chatbot in this context (acceptability), and whether the chatbot would respond to users as instructed (chatbot fidelity).

## Methods

### Overview

Participants were recruited in January 2025 and February 2025 by posting a Human Intelligence Task (HIT) in Amazon Mechanical Turk (MTurk) for smokers who were willing to review health messages. MTurk is a marketplace run by Amazon that connects workers willing to do tasks for businesses, researchers, and others. It allows workers to self-identify as being smokers (and having other characteristics) as part of its registration survey. MTurk workers were able to access the HIT if they were over 18 years of age, lived in the United States, had an HIT approval rate of ≥85%, and self-identified as smokers in the MTurk registration system. In addition, the MTurk workers were eligible if they indicated on our eligibility survey that (1) they had smoked a cigarette in the previous 7 days and (2) they were willing to receive SMS text messages. No requirement was established regarding interest in quitting or motivation to quit. To prevent fraudulent enrollees, participants were excluded if they failed an attention-check question on the screener, a question designed to assess whether the participant was reading the question and unrelated to the subject of quitting smoking (eg, “Pick the response that is a vegetable”).

Eligible MTurk workers provided consent and completed a 30-minute web-based baseline survey, during which they were required to set a quit date within the following week with a default date of Monday. Participants were then enrolled in the BeFree SMS text messaging program, which included instructions on accessing BeFreeBot. Participants completed follow-up surveys 4 weeks after baseline.

### Ethical Considerations

This study was reviewed by the George Washington University Institutional Review Board (NCR235403) and deemed to be exempt. Participants provided written informed consent to be enrolled in the study via Qualtrics (Qualtrics International Inc). The consent form informed participants that conversations with the chatbot would be reviewed by study staff and would be monitored twice a week for the duration of the study. The surveys were stored on a secure, password-protected platform, and only authorized research staff had access to survey responses. Participants received a US $20 gift card for completing the baseline survey and US $25 for completing the 2- and 4-week follow-up surveys (the 2-week results are not presented in this paper). As a precautionary measure, chatbot conversations were reviewed by study staff 2 to 3 times per week for the presence of any alarming comments by the chatbot (eg, chatbot suggestions that may result in harm).

### Intervention

#### BeFree

BeFree is an automated SMS text messaging program for smoking cessation and modeled on previous programs that have been found to double the chances of quitting, such as Text2Quit and SmokefreeTXT [5-7]. BeFree sends out 1 to 2 messages per day timed around each participant’s chosen quit date and continuing for 30 days after enrollment. Message content includes an overview of the quitting process and triggers, behavioral tools and techniques to resist cravings, information on pharmacotherapy, information on how to maintain abstinence and respond to slipups, and general motivational messaging aimed at increasing self-efficacy. Users were given on-demand keywords for help with cravings (“CRAVE”) and for relapse and resetting their quit date (“SMOKED”).

To promote engagement with BeFreeBot (see the following section), for the first 7 days and then every 5 days after that, messages from BeFree ended by prompting the user to reach out and text BeFreeBot on a different phone number for more information on that subject. For example, one message from BeFree read the following: “BeFree: Remember, every day you’re adding benefits for your health, wallet and your future. Text BeFreeBot and share your challenges: [Phone number].” At any point, participants had the option to end all messaging by texting “STOP” to either number.

#### BeFreeBot

BeFreeBot was a revised version of an earlier chatbot created by our team called BeFreeGPT [18]. BeFreeBot was created using a ChatGPT Plus account using GPT-4o-2024-11-20, which has training data up to October 2023, and the Assistants application programming interface [22]. The Assistants application programming interface feature allows for a simplified process of creating a chatbot. It allows for the provision of instructions to the chatbot in prose (vs code) and for the uploading of documents that form a knowledge base that has priority over the model’s own existing knowledge base [23].

**Figure 1:**
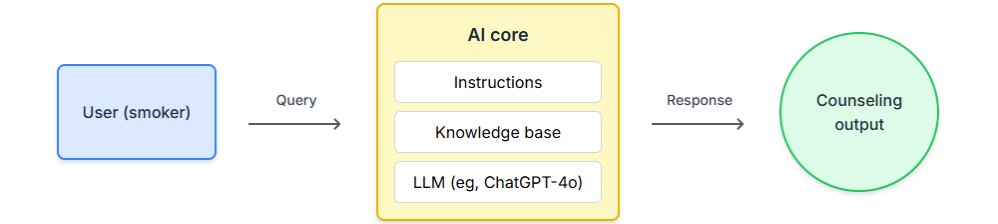
Overview of BeFreeBot’s inputs and outputs.

We provided instructions to the chatbot to act like a smoking cessation counselor, be empathetic, ask follow-up questions, and provide smoking cessation advice based on the documents provided in its knowledge base. Additional instructions to the chatbot included directions to introduce itself before answering the first question (ie, say, “Hi I’m BeFreeBot, an AI counselor to help you quit smoking...”), instructions to make responses less than 50 words long, and a directive to limit conversations to the topic of quitting smoking. The documents uploaded to its knowledge base were (1) the USPSTF recommendation statement *Interventions for Tobacco Smoking Cessation in Adults, Including Pregnant Persons* [3]; (2) *Clearing the Air: Quit Smoking Today*, a guidebook developed by the National Cancer Institute on how to quit smoking [24]; and (3) Food and Drug Administration (FDA)–approved and FDA-cleared cessation products [25]. Figure 1 shows a schematic of BeFreeBot’s inputs and outputs.

**Figure 2:**
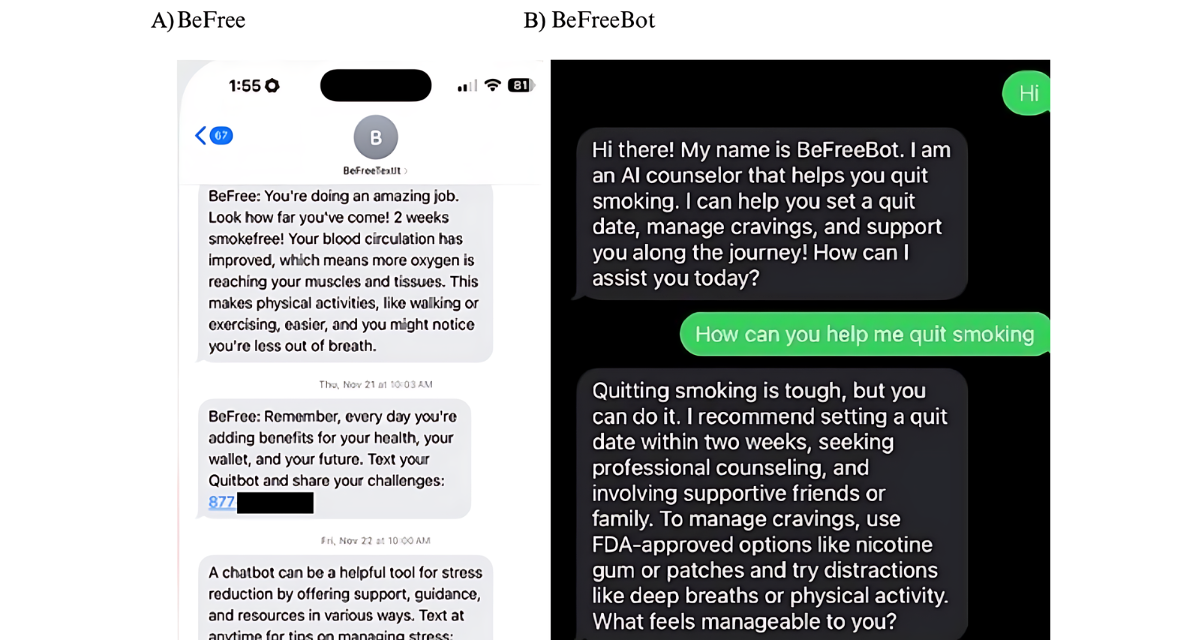
Example of a text message from (A) BeFree and (B) BeFreeBot.

BeFreeBot differed from BeFreeGPT [18] in that it had additional instructions to increase its engagement with participants (eg, instructions to ask follow-up questions) and provide additional counselor-like empathy in its responses and a list of FDA-approved cessation products. In this study, participants could access BeFreeBot through SMS text messages and receive instant SMS text message responses in natural language to any query. This novel format was developed to simulate texting a live counselor who was available 24/7. At enrollment, BeFreeBot reached out to participants to introduce itself. In addition, at 2 weeks after enrollment, a check-in message was sent to each user from BeFreeBot that read the following: “Hi! It’s BeFreeBot. Just checking in to hear how your quit attempt is going. Let me know if there are any challenges I can help you with?” Because of the experimental nature of BeFreeBot and the concern that interactions with it could be harmful to participants, all interactions between BeFreeBot and participants were reviewed by study staff twice a week for the duration of the trial to detect any potential problems (See Figure 2 for an example of messages with BeFree and BeFreeBot).

### Measures

#### Overview

Demographic information (eg, age, gender, race and ethnicity, and educational level) and smoking characteristics were assessed on the baseline survey. Smoking characteristics included the average number of cigarettes smoked per day, whether they had had a past-year quit attempt (yes or no), nicotine dependence measured using the Fagerstrom Test for Nicotine Dependence [26], and an assessment of other tobacco products used.

#### Primary Outcome

The primary outcome for this study was BeFreeBot feasibility, which was operationalized as BeFreeBot engagement. Successful engagement was defined a priori as whether participants engaged with BeFreeBot by texting it a question or concern at least one time. The threshold of at least one text to the chatbot was chosen to reflect that this behavior, texting the chatbot, was somewhat complicated for users. Due to technical limitations of the system, users could not simply reply via text to the originating BeFree message to access the chatbot. Instead, they had to text the chatbot by texting *a different number*, requiring an additional step. Other metrics of engagement were the total number of texts sent to BeFreeBot by participants and the number of days of texting BeFreeBot. The data for these measures came from BeFreeBot computer records, which were accessed via the study account on Twilio (Twilio Inc), the SMS text messaging system.

#### Secondary Outcomes

A secondary outcome was the acceptability of BeFreeBot to participants. This outcome was assessed through participants’ ratings of the program on the 4-week follow-up survey. This included indicators on a 5-point Likert scale of agreement (eg, 1=“not at all”; 5=“completely”), with statements about satisfaction with BeFreeBot, the helpfulness of BeFreeBot (“I think that BeFreeBot is competent and effective in helping people quit smoking”), their trust in responses from BeFreeBot, and whether they would recommend BeFreeBot to a friend. A rating of 4 or 5 was coded as “in agreement,” and the percentage that agreed was calculated. Open-ended questions soliciting suggestions for improving the program were also asked to participants. One item also assessed the acceptability of the BeFree program overall (ie, the outgoing automated texts and the chatbot).

#### Exploratory Outcomes

Also of interest for exploratory purposes were smoking-related outcomes measured at the 4-week follow-up: smoking status using 7-day point prevalence abstinence and past-month quit attempts, defined as not smoking for at least 24 hours because of trying to quit. In addition, changes in the number of cigarettes smoked per day from baseline to follow-up were measured.

#### Chatbot Fidelity

Finally, of interest was an assessment of chatbot fidelity, that is, whether the chatbot responses to participants were in accordance with its instructions. Measures of chatbot fidelity included whether the chatbot followed its instructions in the following ways: (1) introduced itself before answering the first question, (2) provided responses that were less than 50 words long, (3) offered responses in clear language, (4) showed markers of empathy in more than half of the responses to a participant, and (5) ended the responses to a question with a follow-up question to stimulate engagement. Related to smoking cessation guidelines [3], the chatbot was assessed for whether it (1) provided information to each participant in the course of the conversation on how to handle cravings using behaviors (eg, at least one strategy provided on how to handle cravings, such as by replacing smoking with deep breaths and physical activity, distracting the hands and mind, and changing the routine), (2) provided a recommendation to each participant in the course of the conversation to seek out professional counseling (eg, a physician or a quitline), (3) provided information to each participant on seeking out peer social support, (4) recommended to each participant to consider using NRT or non-NRT prescription medications, and (5) stayed on the topic of quitting smoking (even when participants brought up other topics). In addition, the chatbot responses were assessed for not providing misinformation, defined as advice on quitting smoking outside of USPSTF guidelines [3]. Finally, the chatbot was assessed for not having technical errors (eg, not providing a response at all or giving an error message).

### Analysis

Means and proportions were calculated for outcomes including engagement (feasibility), acceptability, and smoking-related outcomes. Dichotomous smoking outcomes (eg, 7-day point prevalence abstinence) were calculated through self-report at 4 weeks and using missing data imputed as smoking. Responses to the open-ended item soliciting suggestions for improving the program were grouped thematically and summarized.

To conduct the analysis of BeFreeBot instruction adherence (fidelity), the BeFreeBot conversations for the 16 participants who engaged with BeFreeBot were exported from Twilio and analyzed across adherence items. Coders were trained to understand our measures of chatbot fidelity, most of which had been used in a previous study that examined chatbot adherence to the USPSTF guidelines [18]. Two independent coders (CW and AY) were instructed to read the complete conversation transcript for an individual and chatbot and then make inferences about the items of interest and code the items. This was done for all the aforementioned items except for the chatbot introducing itself and providing responses less than 50 words long. Where discrepancies arose, a third coder (TCW) served as a tiebreaker. Across 16 participant conversations and 9 items, the overall agreement was 86%, and the average κ was 0.68 (SD=0.23) (good or substantial agreement) [27]. The items related to whether the chatbot introduced itself (yes or no) and response length of <50 words were coded by only 1 coder (AY) as these items did not have a subjective component.

## Results

### Background

A total of 87 MTurk workers completed the eligibility survey. Of those 87 workers, 57 (66%) were ineligible (n=25, 44% because they had not smoked in the previous 7 days; n=24, 42% because they were not willing to receive SMS text messages; n=5, 9% because they were duplicates; and n=3, 5% because they did not pass the attention check question [categories not mutually exclusive]). After consenting, of the remaining 30 participants, 7 (23%) additional participants were excluded (n=6, 86% because they did not complete the baseline survey and enrollment procedures after consent and n=1, 14% because they had an invalid phone number), leaving 23 (77%) participants enrolled in the study. At 4 weeks, 83% (19/23) of the participants completed the follow-up survey.

### Baseline Demographic and Smoking Characteristics

On average, participants were aged 50 (SD 12.4) years, with an approximately even distribution between male and female individuals and most having attended some college or higher (19/23, 83%; Table 1). Participants were largely of White race (20/23, 87%), and 13% (3/23) were of Hispanic origin. The average number of cigarettes smoked per day at baseline was 11.2 (SD 6.8), the average Fagerstrom Test for Nicotine Dependence score was 3.6 (SD 2.6), and just over a third of the participants (8/23, 35%) had made a quit attempt in the previous year. Other tobacco products used over the previous week included e-cigarettes (7/23, 30%) and, to a lesser extent, other combustible products (eg, cigars; 2/23, 9%) and smokeless products (2/23, 9%).

**Table 1:** Baseline demographic and smoking characteristics of the participants (N=23).

Characteristic		Values
Age (y), mean (SD)		50.0 (12.4)
**Sex, n (%)**		
	Male	12 (52)
	Female	11 (48)
**Hispanic, Latino, or Spanish origin, n (%)**		
	Yes	3 (13)
	No	20 (87)
**Race, n (%)**		
	Black or African American	3 (13)
	White	20 (87)
	Other	0 (0)
**Educational level, n (%)**		
	High school or lower	4 (17)
	Some college or trade school	9 (39)
	College graduate or higher	10 (43)
**Employment status, n (%)**		
	Unemployed	2 (9)
	Employed part time	2 (9)
	Employed full time	19 (83)
**Marital status, n (%)**		
	Single	5 (22)
	Married	9 (39)
	Other (living with partner, divorced, separated, or widowed)	9 (39)
Cigarettes per day, mean (SD)		11.2 (6.8)
FTND^a^ score (0-10), mean (SD)		3.6 (2.6)
Smoking within 30 minutes of waking, n (%)		14 (61)
Quit attempt in the previous year, n (%)		8 (35)
**Other tobacco products used in the previous week, n (%)**		
	e-cigarettes	7 (30)
	Other combustible products (cigars, cigarillos, pipes, bidis, or hookah)	2 (9)
	Smokeless products (dissolvable or chewed tobacco)	2 (9)

^a^FTND: Fagerstrom Test for Nicotine Dependence.

### Participant Engagement, Perceptions, and Smoking-Related Outcomes

Most participants texted at least one question to the BeFreeBot (16/23, 70%; Table 2). In all, there were 661 text exchanges between BeFreeBot and the participants, with 333 (50.4%) texts sent to BeFreeBot by the participants, or 14.5 (SD 23.6) texts per participant on average, and 328 (49.6%) replies from BeFreeBot, or 14.3 (SD 23.0) replies per participant on average. Of the 16 participants who texted BeFreeBot at least once, 12 (75%) sent between 1 and 29 texts, and 4 (25%) sent between 30 and 96 texts. Text conversations with BeFreeBot spanned 3.9 (SD 4.4) days on average, with half of participants (8/16, 50%) engaging in conversation only during the first 2 weeks of enrollment in the program. In total, 9% (2/23) of the participants unsubscribed from the program using the keyword “STOP” before the end of the 4-week trial.

**Table 2:** Participant engagement, perceptions, and smoking-related outcomes (4 weeks; N=23).

			Values
**Engagement**			
	Texted the chatbot at least once, n/N (%)	16/23 (70)	
	Number of texts sent to BeFreeBot, mean (SD; range)	14.5 (23.6; 0-96)	
	Responses sent by BeFreeBot, mean (SD; range)	14.3 (23.0; 0-92)	
	Days texting BeFreeBot, mean (SD)	3.9 (4.4)	
**Acceptability, n/N (%)**			
	Highly satisfied with the program overall	13/19 (68)	
	Highly satisfied with BeFreeBot	13/18 (72)	
	BeFreeBot was helpful	11/19 (58)	
	BeFreeBot responses were easy to understand	16/17 (94)	
	BeFreeBot could answer all questions	12/17 (71)	
	Would recommend BeFreeBot to a friend	13/18 (72)	
	Trusted responses from BeFreeBot	12/17 (71)	
	Preferred an AI^a^ counselor over a human one	7/19 (37)	
**Smoking-related outcomes**			
	Self-report of no smoking in the previous 7 days, n/N (%)	7/19 (37)	
	Self-report of no smoking in the previous 7 days (missing imputed as smoking), n/N (%)	7/23 (30)	
	Change in cigarettes smoked per day, mean (SD)	4.5 (7.8)	
	Past-month quit attempt (yes), n/N (%)	17/19 (90)	

^a^AI: artificial intelligence.

Participants generally reported overall satisfaction with the BeFree program (including BeFreeBot; 13/19, 68%) and with BeFreeBot specifically (13/18, 72%). With regard to BeFreeBot, most participants reported that the BeFreeBot responses were easy to understand (16/17, 94%), that they found the bot helpful for quitting smoking (11/19, 58%), that it could answer all questions about quitting (12/17, 71%), and that they would recommend the program to a friend (13/18, 72%). Most participants stated that they trusted the responses from BeFreeBot (12/17, 71%), although less than half endorsed the idea that they preferred an AI counselor over a human one (7/19, 37%). At the end of the study, 90% (17/19) of the participants reported a quit attempt in the previous month, and 30% (7/23) reported being abstinent from smoking for the previous 7 days. Participants reduced their smoking by 4.5 (SD 7.8) cigarettes per day on average. The most common suggestions for improving the program were enabling direct replies to texts to access BeFreeBot (eg, not requiring having to text a different number to access BeFreeBot) and having BeFreeBot have greater conversational depth and less small talk.

### BeFreeBot Fidelity

An analysis of transcripts of BeFreeBot responses to participants revealed that, generally, BeFreeBot functioned as directed. Participant queries covered a variety of topics, including how to quit smoking; medications for quitting; how to handle specific stressors; and how to use meditation, food, or music to manage cravings. On the basis of the coding analysis, responses (n=328) used clear language (328/328, 100%), had markers of empathy (328/328, 100%), and promoted engagement with additional follow-up questions to participants (13/16, 81%, See Table 3). Most responses to participants included a recommendation to seek out professional counseling (13/16, 81%) or find peer support (11/16, 69%), recommended considering the use of FDA-approved medications (eg, NRT; 14/16, 88%), and provided information on how to manage cravings (13/16, 81%). Responses stayed on the topic of smoking cessation counseling (324/328, 99%) and did not include information that contradicted the USPSTF guidelines (328/328, 100%) or any alarming responses (eg, a response that would result in harm). Responses were mostly at the appropriate length of less than 50 words (309/328, 94%), and there were few technical errors (10/328, 3%). In the case of technical errors, these consisted of the chatbot replying “error” or not replying at all. One area in which instructions were not followed regarded BeFreeBot introducing itself to participants when first accessed. This occurred less than half (7/16, 44%) of the time.

**Table 3:** Results on whether BeFreeBot functioned as directed.^a^

	Instances, n/N (%)
Responses used clear language	328/328 (100)
Showed empathy in more than half of the responses to each participant^b^	16/16 (100)
Promoted engagement after answering the initial question^b^	13/16 (81)
Recommended professional counseling to each participant^b^	13/16 (81)
Recommended FDA^c^-approved medications to each participant^b^	14/16 (88)
Recommended seeking out peer social support to each participant^b^	11/16 (69)
Provided information on how to manage cravings to each participant^b^	13/16 (81)
Responses stayed on the topic of smoking cessation	324/328 (99)
No information that contradicted USPSTF^d^ guidelines or no hallucinations	328/328 (100)
Introduced itself before answering the first question^b^	7/16 (44)
Responses were <50 words long	309/328 (94)
Responses had a technical error	10/328 (3)

^a^328 total BeFreeBot responses or messages across 16 participant conversations.

^b^Analyses conducted at the participant level.

^c^FDA: Food and Drug Administration.

^d^USPSTF: US Preventive Services Task Force.

## Discussion

### Principal Findings

ChatGPT and other LLM chatbots, while increasingly used by the public, have been understudied in the context of smokers trying to quit. In this study, we found that an LLM chatbot integrated into an SMS text messaging program and accessed through SMS text message was found to be feasible and acceptable to smokers for quitting. While participants were required to text a new number to access BeFreeBot, a somewhat cumbersome procedure, most participants (16/23, 70%) managed this and texted BeFreeBot at least once with a question. Participants generally expressed satisfaction with BeFreeBot (13/18, 72%), stating that it was easy to understand (16/17, 94%) and they trusted its responses (12/17, 71%). Quitting outcomes were promising, with most participants (17/19, 90%) making a quit attempt and almost one-third of the participants (7/23, 30%) reporting that they had quit.

The frequency of participant engagement with BeFreeBot is noteworthy given that texting an LLM chatbot is a novel format whose feasibility has not yet been assessed. Not only did we find that this format was feasible, but we also found high levels of engagement. While, on average, participants texted BeFreeBot 14.3 (SD 23.0) times, the range was considerable, with a couple of participants (2/23, 9%) texting BeFreeBot over 50 times (and receiving over 50 texts back). This level of engagement is encouraging for SMS text messaging programs as automated SMS text messaging programs generally offer short and formulaic forms of engagement (eg, texting the keyword “CRAVE” for a tip on managing cravings) [28]. Indeed, the chatbot’s ability to understand natural text and provide personalized responses to queries on topics such as specific stressors appears to have enabled a longer form of engagement on a given topic and possibly a deeper form of engagement than that with previous formulaic SMS text messaging programs. As program engagement has been linked to health behavior change [28,29], chatbots may be effective by boosting engagement. Studies are needed to further explore this possibility, including exploring the role that literacy may play in text-based chatbot engagement.

Our study also identified areas for design improvements. While text engagement was frequent, it was also short-lived. Conversations with BeFreeBot took place, on average, on 3.9 (SD 4.4) of 30 possible days of the study, with half of participants only engaging in conversation during the first 2 weeks after enrollment. This aspect of engagement could be improved by providing participants with additional prompts to engage with the chatbot and suggested conversation starters (eg, “Ask BeFreeBot about how to deal with your biggest trigger for smoking”). Indeed, we sent the last engagement prompt at 2 weeks, and such prompts could be extended in time for a longer period. In addition, the most common open-ended suggestion from participants was enabling direct replies to texts to access BeFreeBot (eg, not requiring having to text a different number to access BeFreeBot). Future efforts will be aimed at identifying a technical solution so that BeFree and BeFreeBot can be integrated into 1 phone number to improve the user experience. Indeed, as the technology is quickly evolving, new design possibilities will also be explored. These include possibilities that go beyond SMS text messaging and allow for the inclusion of generative images and videos and the use of voice instead or in addition to text, which might address literacy concerns.

Another finding of our study is that our chatbot, for the most part, adhered to our instructions and sent responses that followed them. An analysis of transcripts of the BeFreeBot responses to participants revealed that BeFreeBot stayed on the topic of quitting smoking even when participant queries veered outside of this topic. The chatbot also showed at least minimal levels of empathy and promoted engagement by asking follow-up questions, as well as adhering to recommendations consistent with USPSTF guidelines [3]. This preliminary finding is important given concerns published in the literature about the presence of hallucinations and misinformation in chatbot responses [30] and general concerns about the ethics of having a generative AI chatbot as a counselor or coach [31,32].

Indeed, to achieve the observed levels of adherence, our team dedicated considerable effort before the trial to validating, refining, and revising our instructions [18]. Still, some areas for improvement were identified, such as in following the direction to introduce itself during the first interaction with a participant, which the chatbot did less than half of the time. In the future, BeFreeBot instructions will be further refined to emphasize following them and ensure that they are followed for a greater variety of responses.

The strength of this study is that it represents one of the first to study a specialized ChatGPT-based chatbot with people who smoke and were willing to set a quit date. It is also the first study to our knowledge of a chatbot accessed through an automated SMS text messaging program. Results show that the chatbot and its SMS text messaging interface were feasible and mostly appreciated by smokers. These findings are important because ChatGPT has potential as a scalable tool in reaching the public with help for quitting smoking. Our findings suggest that such adaptations hold potential for supporting smoking cessation efforts.

The limitations of our study are that it was a pilot study with no control group. The small sample size implies that the chatbot only received a small number of queries, and the possibility remains that additional queries could have led the chatbot off topic or to diverge from its instructions in other ways. Future research that includes a larger sample, as well as a comparison group, will be able to test whether chatbots offer benefits for quitting beyond those of standard digital programs. Another limitation of this study is its generalizability. Apart from the small sample size, participants recruited on MTurk represent a specific type of employed person. By the nature of the MTurk work, workers likely have access to a PC and work at a desk. In our sample, participants were predominantly of White ethnicity and college educated. Future research is needed to demonstrate effects among a more diverse sample, including individuals with low levels of education and those with low literacy levels as these characteristics may impact engagement with a text-based program. While we created a crude metric for measuring chatbot adherence to instructions, especially in the measurement of empathy, more nuanced metrics could be developed. Furthermore, additional procedures could be put in place to ensure that the fidelity assessment team is independent from the development team, which in our pilot had some overlap and could introduce bias. Finally, our measure of acceptability could be improved by using a validated scale.

### Conclusions

In conclusion, an LLM chatbot integrated into an SMS text messaging program and accessed through SMS text message was found to be feasible and acceptable to smokers. Future iterations of the program will be aimed at increasing engagement with BeFreeBot, facilitating conversations that span more days, and rigorously testing the program’s efficacy in producing long-term benefits for quitting smoking. As the program is scaled to larger samples with more diverse backgrounds, a key consideration will be how to ensure that the LLM chatbot continues to provide evidence-based information and benefits to all groups of users.

## Abbreviations

AI: artificial intelligence
FDA: Food and Drug Administration
HIT: Human Intelligence Task
LLM: large language model
MTurk: Amazon Mechanical Turk
NRT: nicotine replacement therapy
SARAH: Smart Artificial Intelligence Resource Assistant for Health
USPSTF: United States Preventive Services Task Force

## Data Availability

The datasets generated or analyzed during this study are available from the corresponding author on reasonable request.
